# Amino Acid Betaxanthins: Absorption, Fluorescence, And Stability

**DOI:** 10.1021/acs.jnatprod.5c00419

**Published:** 2025-06-20

**Authors:** Larissa C. Esteves, Amanda C. Pinheiro, Caroline O Machado, Nathana B. L. Pettigiani, Ana Clara B. Rodrigues, Masahiko Taniguchi, Jonathan S. Lindsey, Erick L. Bastos

**Affiliations:** † Department of Fundamental Chemistry, Institute of Chemistry, 28133 University of São Paulo, 05508-000 São Paulo, São Paulo, Brazil; ‡ Department of Chemistry, 6798 North Carolina State University, Raleigh, North Carolina 27607, United States

## Abstract

Betaxanthins are natural pigments responsible for the vivid yellow coloration and intrinsic fluorescence of flowering succulent plants within the order *Caryophyllales*. Though less extensively studied than other plant pigments classes, betaxanthins hold potential for application as safe food dyes, solar cell absorbers, antioxidants, and genetically encodable fluorophores. Herein, we report the absorption spectra, fluorescence properties, and hydrolysis rate constants of 24 distinct betaxanthins obtained by semisynthesis from betalamic acid and a variety of amino acids. The molecular library includes all known derivatives from amino acids present in plants and fungi, as well as cysteine-betaxanthin (which remains undetected in nature) and a selection of model non-natural analogues. Structure–property relationships were examined to contextualize the spectroscopic data. Medium effects on the spectral properties of both betaxanthins and their biosynthetic precursor, betalamic acid, are discussed in terms of light energy dissipation, supporting a proposed photoprotective role for these secondary metabolites *in vivo*. All spectral data have been made accessible in a PhotochemCAD absorption/fluorescence spectral database, enabling streamlined analysis and quantification.

## Graphical abstract

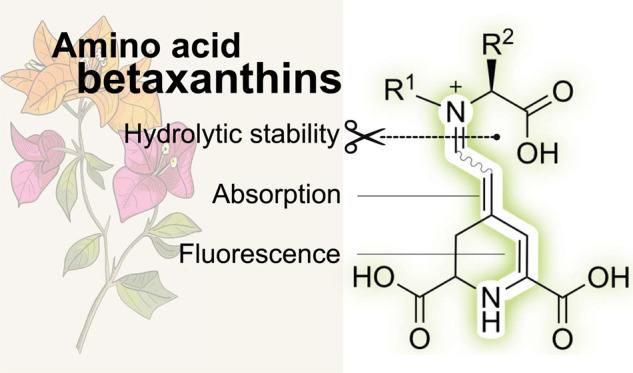

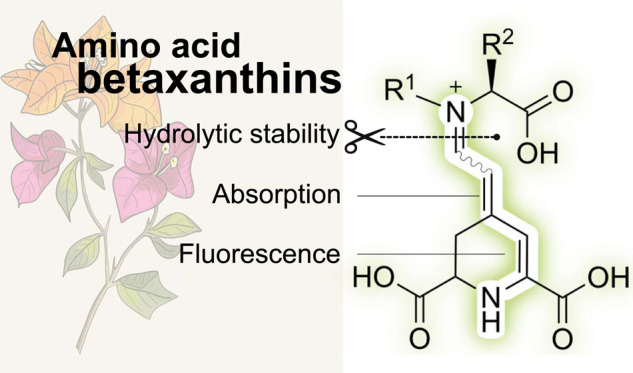

Betalains are natural pigments that contribute color diversity in plants.^1^ Their biological functions involve visual signaling^2^ and protection against biotic and abiotic stresses.^3,4^ The biosynthesis of betalains from l-tyrosine and the properties of this class of natural products are of much interest,^5^ not only to increase knowledge about the natural world but also due to their potential application as biocompatible dyes,^6,7^ redox mediators,^8,9^ and genetically encodable colors and reporters, fluorescent markers, and therapeutic agents.^10–14^

The characteristic red color of beetroots (*Beta vulgaris* L.; Latin *be̅ta* means beet) is due to the combination of magenta betacyanins and yellow betaxanthins, the two subclasses of betalains. Changes in the relative proportion of betaxanthins and betacyanins produce colors that vary from yellow and magenta, passing through shades of orange to red.^4^ The colors of the iconic fly agaric toadstool [*Amanita muscaria* (L.) Lam.][Bibr ref15] and of some fungi of the genera *Amanita*, *Hygrocybe*, and *Hygrophorus* are partly due to their betalain content.^16–18^ Flowers of the yellow varieties of bougainvillea (*Bougainvillea* sp.), four o’clock (*Mirabilis jalapa* L.), purple ice (*Lampranthus productus* N.E.Br), rose moss (*Portulaca grandiflora* Hook.), and plumed cockscomb (*Celosia argentea* L.) show green fluorescence when their vacuolar betaxanthin pigments absorb blue light.^19–21^ Because betaxanthins are poor emitters in solution (fluorescence quantum yield, ϕ_Fl_ < 1%),^22–27^ it has been suggested that the potential function of fluorescence in these plants is to enhance color glow, instead of acting as an independent signal.^28^

Betalains are water-soluble chiral imines or iminium salts originating from the reaction of betalamic acid and nitrogen nucleophiles, such as amines and amino acids.^29^ All betalains contain a pentamethinium cyanine chromophore (Figure 1).^30,31^ Natural betacyanins are derivatives of cyclo-DOPA, which contains a catechol auxochrome group that increases the molar absorption coefficient (*ε*) and shifts the absorption maxima (λ_Abs_) toward longer wavelengths compared to betaxanthins; *ε* ^540 nm^(betacyanins) ∼ 65,000 L mol^–1^ cm^–1^ vs *ε* ^480 nm^(betaxanthins) ∼ 48,000 L mol^–1^ cm^–1^.^4,32^ Betacyanins absorb green light, are virtually nonfluorescent, and can quench the fluorescence of betaxanthins.^19^

**Figure 1:**
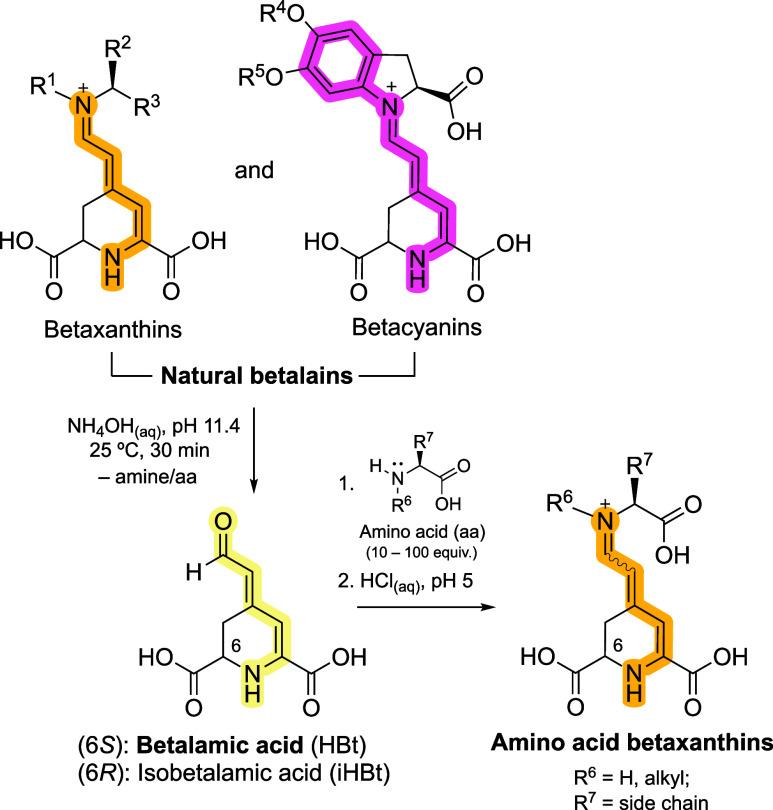
Betalamic acid, obtained by alkaline hydrolysis of natural betalains, is used in the semisynthesis of amino acid betaxanthins. Amino acid-derived betaxanthins are often obtained as mixtures of diastereomers due to double-bond isomerization within the pentamethine cyanine system and changes in the absolute configuration at the C6 position, which bears a relatively acidic α-hydrogen atom. Since betalamic acid is biosynthesized from l-tyrosine, the absolute configuration at C6 in natural betaxanthins is *S*;^33^ however, isomerization to the 6*R* (*iso*) form has also been reported.^34^ In addition, the conformational flexibility of the dihydropyridine ring can give rise to carboxy-axial and carboxy-equatorial isomers.^35^

Betaxanthins derived from the twenty-two genetically encoded (proteinogenic) amino acids, except for l-cysteine, L-selenocysteine, and L-pyrrolysine, have been found in plants and fungi (Figure 2).^36–46^ These compounds have been obtained in the mg-scale by semisynthesis or biotechnological methods;^15,20,47^ however, literature information concerning their absorption and fluorescence properties is fragmented and scarce.^48^ Their absorption spectra have been most frequently acquired by HPLC/PDA analysis under specific chromatographic conditions,^29,45,49–54^ e.g., acidified water/MeCN mixtures, and heretofore there has been no systematic report on the photophysical properties of amino acid betaxanthins.

**Figure 2:**
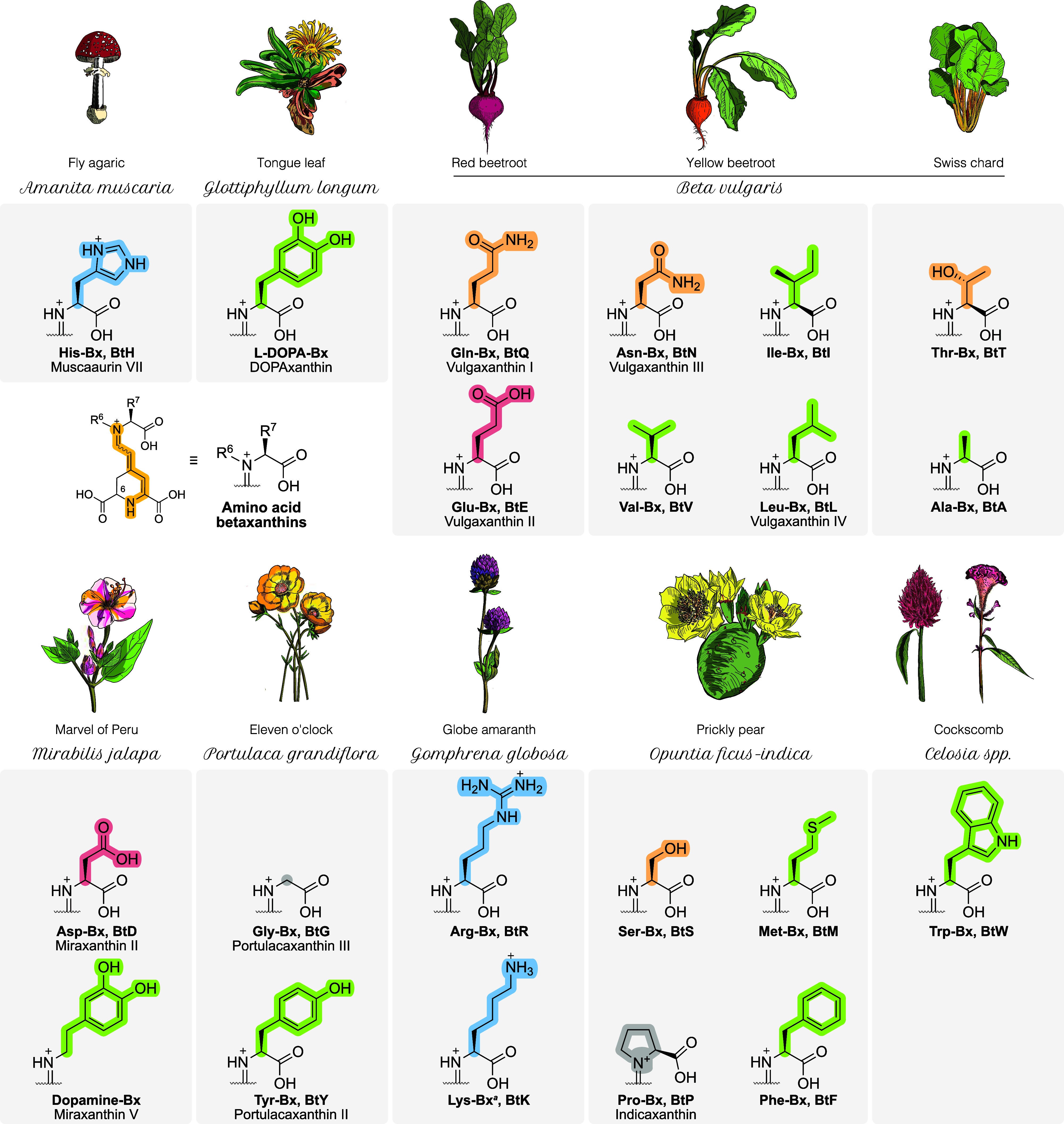
Amino acid betaxanthins and the first-reported species of origin. Specimen illustrations (by E.L.B.) are simplified representations and not anatomically accurate. ^*a*^ Because the exact structure of natural Lys-Bx in *G. globosa* is unknown, the Lys residue is linked by its α-amino group for consistency, although we also studied the ε-Lys-Bx. Side chains are color-coded based on their characteristics: blue for positively charged, red for negatively charged, orange for polar uncharged, green for hydrophobic, and gray for special cases.

We previously described the semisynthesis of high-purity betaxanthins derived from all proteinogenic amino acids, except selenocysteine and L-pyrrolysine, and the characterization of their antioxidant properties.^55^ Here, we report the spectral features of amino acid betaxanthins, including the wavelengths of maximum absorption, excitation, or fluorescence (λ_Abs_, λ_Ex,_ or λ_Fl_, respectively), molar absorption coefficient (*ε*), fluorescence quantum yield (ϕ_Fl_), and brightness, which is taken as *ε x ϕ*_Fl_. These data, along with insights into the persistence of betaxanthins in aqueous media, were analyzed in terms of structure–property relationships, contributing to a deeper understanding of this class of secondary metabolites found in plants and fungi. All photophysical parameters and spectra have been compiled into a dedicated database within PhotochemCAD,^56,57^ enabling comparisons with the emission properties of other betaxanthin-containing biofluorescent species and supporting future studies on betalains across biological systems.

## Results and Discussion

### Absorption and Fluorescence Spectra of Amino Acid Betaxanthins

Betaxanthins were prepared using an environmentally benign method.^55^ The absorption spectra of all naturally occurring derivatives show similar features, with maxima ranging between 471–486 nm and a shoulder at approximately 450 nm (Figure 3 and Table 1). Cysteine-betaxanthin absorption and emission spectra are shifted to longer wavelengths.

**Figure 3:**
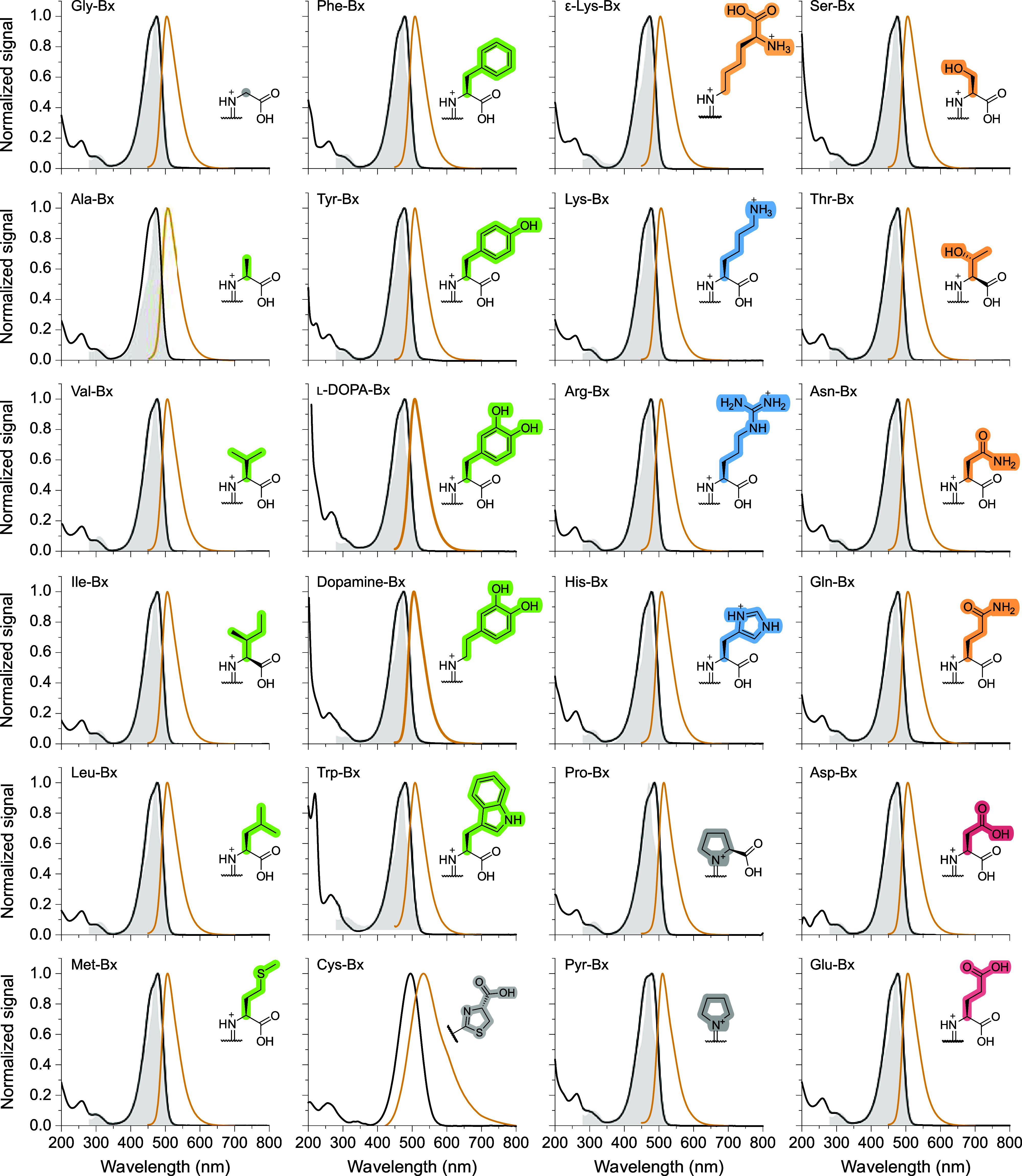
Absorption (black line), excitation (gray filling, λ_Fl_ = 540 nm; λ_Fl_^Cys‑Bx^ = 550 nm), and fluorescence (orange line, λ_Ex_ = 430 nm; λ_Ex_^Cys‑Bx^ = 455 nm) spectra of betaxanthins in water, at 25 °C. Amino acids are presented in the fully protonated form for convenience; side chains are color-coded based on their characteristics: blue for positively charged, red for negatively charged, orange for polar uncharged, green for hydrophobic, and gray for special cases.

**Table 1:** Absorption and Fluorescence Properties of Amino Acid Betaxanthins and Other Derivatives of Interest

Bx	λ_Abs_ (nm)	*ε* ^a^ (10^4^ L mol^–1^ cm^–1^)	λ_Ex_ ^b^ (nm)	λ_Fl_ ^b^ (nm)	Stokes shift, Δλ (nm, [cm^–1^]^c^)	*E*_S_ (kJ mol^–1^)	ϕ_Fl_ (10^–3^)	Brightness^d^ε × ϕ_Fl_	*k*_obs_ ^e^ (s^–1^)
Ala-Bx	473	5.19	469	505	32, [1340]	245	4.6 ± 0.2	239 ± 10	1.8 × 10^–4^
Arg-Bx	476	5.02	469	507	31, [1285]	244	9.3 ± 0.4	467 ± 20	1.8 × 10^–4^
Asn-Bx	476	5.62	469	507	31, [1285]	244	7.4 ± 0.4	416 ± 22	2.4 × 10^–4^
Asp-Bx	476	5.66	470	507	31, [1285]	244	5.1 ± 0.3	289 ± 17	2.1 × 10^–4^
Cys-Bx	495	0.73^f^	ND	532	41, [1405]	234	<0.1	<1.0	2.9 × 10^–6^
DA-Bx	474	5.46	469	506	32, [1334]	244	2.6 ± 0.1	142 ± 5	1.2 × 10^–4^
Gln-Bx	476	5.16	469	506	30, [1246]	244	6.5 ± 0.4	335 ± 21	1.8 × 10^–4^
Glu-Bx	475	5.52	469	506	31, [1290]	244	6.2 ± 0.4	342 ± 22	1.2 × 10^–4^
Gly-Bx	473	5.28	469	504	31, [1300]	245	4.6 ± 0.2	243 ± 11	2.7 × 10^–4^
His-Bx	477	4.99	469	507	30, [1240]	243	9.9 ± 0.5	494 ± 25	2.9 × 10^–4^
Ile-Bx	476	5.69	469	505	29, [1206]	244	5.4 ± 0.3	307 ± 17	6.3 × 10^–5^
DOPA-Bx	479	5.65	470	507	28, [1153]	243	3.6 ± 0.2	203 ± 11	1.3 × 10^–4^
Leu-Bx	476	5.21	469	507	31, [1285]	244	6.8 ± 0.4	354 ± 21	1.2 × 10^–4^
Lys-Bx	475	5.41	470	506	31, [1290]	244	7.8 ± 0.4	422 ± 22	1.6 × 10^–4^
ε-Lys-Bx	471	4.83	470	503	34, [1351]	245	4.2 ± 0.2	203 ± 10	9.3 × 10^–5^
Met-Bx	475	5.54	469	508	33, [1368]	244	6.3 ± 0.4	349 ± 22	1.5 × 10^–4^
Phe-Bx	477	5.21	469	508	31, [1279]	243	7.2 ± 0.4	375 ± 21	1.0 × 10^–4^
Pro-Bx	486	5.74	469	513	27, [1083]	239	4.8 ± 0.3	276 ± 17	1.3 × 10^–5^
Pyr-Bx	479	5.01	469	512	33, [1346]	242	1.0 ± 0.1	50 ± 5	1.0 × 10^–5^
Ser-Bx	474	5.45	469	505	31, [1295]	244	5.6 ± 0.3	305 ± 16	2.3 × 10^–4^
Thr-Bx	476	5.35	469	506	30, [1246]	244	5.7 ± 0.2	305 ± 11	1.4 × 10^–4^
Trp-Bx	478	4.77	469	510	32, [1313]	243	0.3 ± 0.1	14 ± 5	7.0 × 10^–5^
Tyr-Bx	477	5.20	469	508	31, [1279]	243	5.9 ± 0.4	307 ± 21	9.2 × 10^–5^
Val-Bx	475	5.15	469	505	30, [1251]	244	6.8 ± 0.3	350 ± 15	6.3 × 10^–5^

a In BRB (40 mmol L^–1^, pH 9.5; relative to betalamic acid, *ε* ^424 nm^ = 26,500 L mol^–1^ cm^–1^).^58^

b λ_Ex_ = 430 nm except for Cys-Bx (λ_Ex_ = 455 nm).

c Although full values in cm^–1^ are reported, the level of uncertainty justifies rounding to three significant figures.

d In L mol^–1^ cm^–1^; although full values are reported, the level of uncertainty justifies rounding to two significant figures.

e Hydrolysis of betaxanthins in phosphate buffer (100 mmol L^–1^, pH 7) at 50 °C.

f Not observed *in vivo*; different spectral profile. See text for details.

The molar absorption coefficient (ε) values of the betaxanthins have been estimated by use of an end point method (Figures S1–S3). The conventional determination of molar absorption coefficients by weighing is particularly challenging for betaxanthins due to their high hygroscopicity. These compounds are typically isolated in small amounts (mg-scale), and they rapidly absorb moisture from the air. As a result, precise and reproducible weighing is difficult, often leading to significant errors in concentration estimation,^48^ as further discussed below. The spectra of all betaxanthins were acquired in water pH 7 and in Britton-Robinson universal buffer solution (BRB) pH 9.5–10.5. In each case, the initial absorption at the λ_Abs_ (pH 7) was assumed to be directly proportional to the concentration of betalamic acid formed upon reaction completion; *ε* ^424 nm^ (HBt) = 26,500 L mol^–1^ cm^–1^.^58,59^ For Cys-Bx, the value of *ε* was determined by monitoring the formation of the betaxanthin instead of its hydrolysis and assumes HBt did not degrade during the measurement. Using this approach gave an average *ε* of 53,000 L mol^–1^ cm^–1^ for amino acid betaxanthins and 7,320 L mol^–1^ cm^–1^ for Cys-Bx. Figure S4 depicts an overlay of the spectra for all compounds.

The fluorescence spectra of amino acid betaxanthins were obtained upon excitation with blue light (430 nm, except for Cys-Bx at 455 nm). All betaxanthins, except for Cys-Bx, have a similar fluorescence spectrum, with emission maximum in the range 503–513 nm (Figure 3 and Table 1). The emission of Pro-Bx, which is derived from an amino acid with a secondary amine group, is slightly shifted to longer wavelengths compared to the other betaxanthins. The average ϕ_Fl_ value of the family of betaxanthins is around 10^–3^ (Table 1), in agreement with the values reported for Dopamine-Bx (miraxanthin V, 3.0 × 10^–3^),^26^ Pro-Bx (indicaxanthin, 4.6 × 10^–3^; 5.3 × 10^–3^)^24,27^, Gln-Bx (vulgaxanthin I, 7.3 × 10^–3^),^25^ and miraxanthin I (Methionine sulfoxide-Bx, 8.4 × 10^–3^);^60^ raw data are presented in Figures S5–S7. Trp-Bx is an exception with a ϕ_Fl_ of (3.3 ± 0.2) × 10^–4^. All amino acid betaxanthins show a Stokes shift between 1100–1400 cm^–1^ and singlet energy (*E*_S_) of approximately 245 kJ mol^–1^. These values can be considered moderate compared to common fluorophores such as fluorescein, rhodamine, and cyanine dyes, which typically show greater spectral overlap and higher self-absorption.^61^ Brightness, a key feature for the application of fluorescent compounds as markers^62^ and for the visualization of biofluorescence, was estimated from the values of *ε* and ϕ_Fl_ of each amino acid betaxanthin. In this assessment, Arg-Bx and His-Bx are the brightest derivatives, whereas the least bright betaxanthins are Dopamine (DA)-Bx, Pyr-Bx, and Trp-Bx (Table 1). Overall, although the excitation spectra (Figure 3, gray-filled curves) exhibit higher resolution, they match the absorption spectra; the wavelength of maximum excitation for emission at 540 nm is nearly the same (469–470 nm) for all betaxanthins. The excitation wavelengths (λ_Ex_) listed in Table 1 vary from the wavelength of maximum absorption to the shoulder at shorter wavelengths.

For comparison, we acquired the absorption and emission spectra of betalamic acid in water at pH 7 and investigated the effect of pH on its absorption properties (Figure 4a). The λ_Abs_ at 428 nm, observed within the pH range of 7–10, was shifted from the 424 nm observed under acidic conditions that is used for quantification purposes.^63^ The fluorescence maximum at 495 nm was at a shorter wavelength compared to that of indicaxanthin (Pro-Bx) and other betaxanthins (Table 1). There was no noticeable change in absorbance within the pH range of 7–11, although at pH 11 an increase in absorption in the UVA region was observed. For indicaxanthin, the absorbance and fluorescence intensity were diminished at pH > 10 (Figure 4b). The opposite effect was observed upon the addition of glycerol as a viscogen; the **ϕ**_**Fl**_ increased from 5.4 × 10^–3^ to 4.6 × 10^–2^ in 90% m/m glycerol in water (Figure 4c).

**Figure 4:**
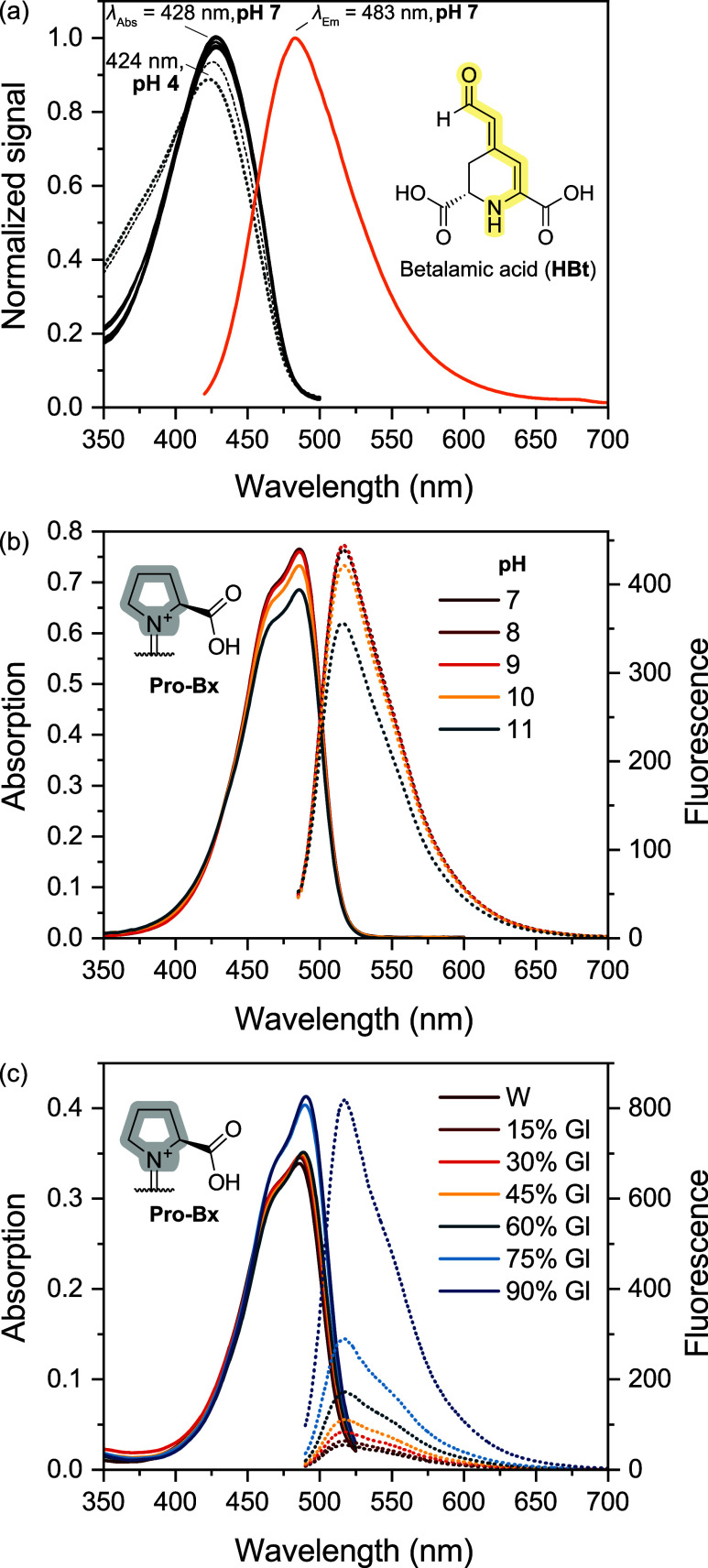
Media effect on the absorption and fluorescence of betalamic acid and indicaxanthin. Fluorescence intensities are shown in arbitrary units and are instrument-dependent; full absorption and emission spectra are presented for direct comparison. (a) Absorption and fluorescence spectra of HBt, λ_Ex_ = 400 nm. Absorption spectra of HBt were acquired at the pH range of 4–10 and normalized to the maximum absorption at pH 7. (b) Absorption and fluorescence spectra of Pro-Bx in solution, pH 7–11. (c) Effect of the glycerol-in-water ratio (% m/m) on the absorption and fluorescence spectra of Pro-Bx in aqueous solution, λ_Ex_ = 470 nm.

### Hydrolytic Stability of Amino Acid Betaxanthins

The semisynthesis and characterization of betaxanthins in aqueous media are challenging due to hydrolytic decomposition. To evaluate hydrolytic stability, the degradation of betaxanthins was monitored in 100 mmol L^–1^ phosphate buffer at pH 7 and 50 °C, allowing observation of more than two half-lives within 6 h. A complete analysis of Gly-Bx is presented in Figure 5 as an example; raw data for all betaxanthins are available in Figures S1–S3 and S8–S11.

**Figure 5:**
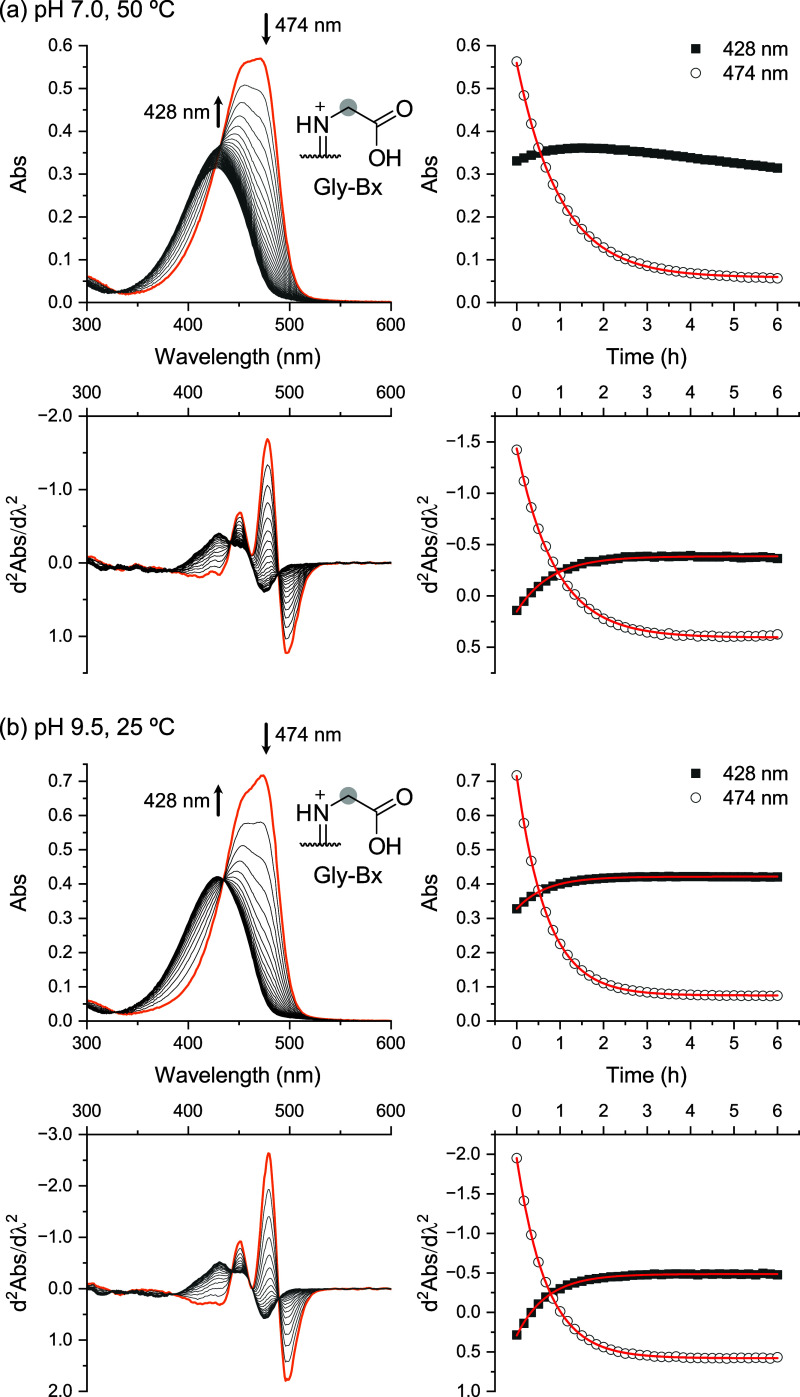
Hydrolysis of Gly-Bx: absorption spectra over time, second-derivative absorption spectra, and corresponding kinetic traces. (a) Hydrolysis under neutral conditions (pH 7, phosphate buffer (PB), 100 mmol L^–1^) at 50 °C was used to determine the hydrolytic stability. (b) Hydrolysis under alkaline conditions (pH 9.5, BRB, 40 mmol L^–1^) at 25 °C was used to determine the molar absorption coefficient using the end point method. Second-derivative data were subjected to Savitzky–Golay smoothing (second order, 20 points in window), but kinetic traces in all spectra use the same wavelengths. Spectra highlighted in orange were acquired at *t*_0_. Red curves are the monoexponential fits of the absorption data. Arrows indicate the directional trend of the band over time.

Time-resolved UV–vis monitoring of Gly-Bx hydrolysis at pH 7 and 50 °C revealed a gradual decrease in the absorption band around 474 nm, accompanied by the appearance of a new band at approximately 428 nm, corresponding to betalamic acid (Figure 5a). Owing to significant band overlap, monitoring absorbance solely at 428 or 474 nm (or even at wavelengths away from these maxima) did not yield reliable kinetic data. First- and higher-order derivative spectra are obtained by analyzing the differential coefficient of absorbance with respect to wavelength; such spectra can track the shifts of the spectral peaks and hence are valuable in the analysis of multicomponent mixtures. Accordingly, second-derivative absorption spectroscopy (d^2^ *Abs*/dλ^2^) was employed to resolve the spectral changes more effectively.^64^ Using this approach, the spectral changes of HBt were resolved, revealing a plateau after 2 h (Figure 5a). Importantly, the observed rate constant (*k*_obs_) for betaxanthin decomposition, determined by fitting an exponential function to the time-dependent changes in both raw absorbance and second-derivative spectra, differed by less than 5%, validating our analytical method.

At pH 7 and 50 °C, most betaxanthins have a maximum half-life of three hours, with His-Bx and Gly-Bx being the least stable betaxanthins against hydrolysis (Table 1, Figures S8–S10). Cys-Bx, Pyr-Bx and Pro-Bx are more stable than the others, for which the absorption decay is insufficient to determine reliable *k*_obs_ values without assuming the infinite-time absorbance for these betaxanthins to be zero. For Cys-Bx, the value determined by extrapolated fitting was approximately 20-times greater than that of Ile-Bx and Val-Bx, and about 3-times greater than that for Pyr-Bx (Figure S11). For comparison, after 6 h, the absorption of Pro-Bx declined by 24% and that of Pyr-Bx only by 19%, while other derivatives exhibited losses between 65% and 92%. Notably, Cys-Bx showed only a 1% decrease in absorbance after 1 h, and just 6% after 6 h.

Base-catalyzed hydrolysis enabled kinetic analysis of betaxanthins at 25 °C; accordingly, end point determination of molar absorption coefficients was performed at pH 9.5. Under these conditions, Gly-Bx hydrolysis resulted in a rise in absorbance at 428 nm (betalamic acid) and a concomitant drop at 474 nm (Gly-Bx) providing suitable kinetic data (Figure 5b). At pH 9.5 and 25 °C, the *k*_obs_ values for the decomposition of all betaxanthin decomposition, except Cys-Bx, correlated with the increase absorbance at 428 nm, confirming the formation of betalamic acid (Figures S1–S3). The saturation profile of the kinetic trace at 428 nm indicated that HBt remained stable during analysis, and that spectral overlap did not compromise kinetic assessment, as corroborated by second-derivative spectral trends.

### Betaxanthins Are Genetically Encodable, Chiral, Pentamethinium Cyanine Colorants

In 1968, Marby and Dreiding coined the term ″betalains″ to describe the red and yellow vacuolar pigments derived from betalamic acid, which were then known to occur only in certain plant species, such as the red beet.^65^ Based on König’s nomenclature for polymethine systems, they identified the ″1,7-diazaheptamethin″ chromophore as responsible for the vivid coloration of betalains.^65,66^ However, the evolving nature of color chemistry has introduced some confusion in naming conventions, prompting ongoing clarification.^67^ Variations on this chromophore’s name, such as 1,7-diazaheptamethine^68^ or 1,7-diazaheptamethinium,^69^ appear throughout the literature, although betalains are now recognized as chiral, water-soluble, pentamethinium cyanine natural colorants.^31^

Polymethine dyes are frequently employed as fluorophores for imaging applications in cells, tissues, and whole organisms due to their straightforward and adaptable synthesis, high molar absorption coefficients, biocompatibility, and their capacity to emit light across the green to near-infrared (NIR) spectrum.^70^ This group of dyes includes several notable classes, such as indoleninium-based carbocyanines (Cy), squaraines, flavylium-based dyes, and coumarin-hemicyanine hybrids. Examples include Indocyanine Green (ICG), a clinically approved NIR dye for vascular imaging, and Cy5 derivatives such as AlexaFluor 647, commonly used in single-molecule localization microscopy.^71,72^ Betalains, particularly betaxanthins, present new opportunities as they are genetically encodable and, unlike many Cy dyes, do not efficiently populate the triplet excited state via intersystem crossing,^23^ making them poor photosensitizers. For many biomedical applications, the *absence of photosensitization* is a desirable feature.

It warrants noting that colorants, including both fluorophores and nonfluorescent substances, can be classified as either dyes or pigments, regardless of whether they are natural or synthetic. Although these terms are often used interchangeably, ideal pigments are defined as substances that are practically insoluble in the medium to which they are applied, requiring additional agents to become attached to a substrate due to the lack of specific affinity.^73^ This distinction is independent of the colorant’s origin. However, depending on the field of study, the classification of natural colorants as dyes or pigments can be unclear.

### Rationale on Their Absorption Spectra and Molar Absorption Coefficients

Betalains are water-soluble compounds that, ironically, are subject to relatively fast hydrolysis.^74^ Since their structural characterization in the early 1960s, important chemical features of betalains are still unknown. These multifunctional compounds contain carboxylic acid groups whose p*K*_a_ values are uncertain but are assumed to be around 3.5, following the reports of Nilsson,^75^ and Piattelli and Minale^76^ for betanin and indicaxanthin, respectively. Also, the cationic nature of the chromophore implies that betalains can exists as zwitterions or salts, and, in the latter case, the counterion identity is relevant for the gravimetric determination of molar absorption coefficients. Therefore, conventional determination of *ε* by weighing is limited by the hygroscopic nature of betalains, lack of information of the exact nature of the solid, presence of impurities and difficulties in obtaining proper crystals, with one exception being for betanin.^77,78^ Consequently, the reported values of the molar absorption coefficients of betalains show considerable variation.^40,75,76,78–80^ For example, the following *ε* values (in L mol^–1^ cm^–1^) have been reported for betanin: 51,000 (542–546 nm),^81^ 62,000 (536–538 nm),^82^ 60,500 (536–538 nm),^83^ 60,000 (538 nm),^84^ 65,000 (535 nm),^78^ 56,234 (536 nm),^40^ and 61,600 (536 nm).^79^ For indicaxanthin, the reported values are as follows: 42,658 (485 nm),^76^ 39,810 (483 nm),^80^ 62,000 (483 nm),^80^ and 50,119 (484 nm).^40^ Indeed, the determination of values for *ε* is often fraught even for compounds that are not hygroscopic, are available in abundance, and are widely used as benchmark compounds. A case in point is for *meso*-tetraphenylporphyrin, where reported values of *ε* range by >10-fold across hundreds of independent literature reports.^85^

For betaxanthins, the *ε* of 48,000 L mol^–1^ cm^–1^ at 480 nm reported by Girod and Zrÿd^32^ is widely used for analytical purposes,^4,29,80,84,86–88^ although it is unclear how this value was estimated. The same group also determined the value of *ε* of betaxanthins and betanin by titrating the amino acids produced upon acidic hydrolysis with aqueous HCl 6 mol L^–1^ for 22 h at 115 °C.^40^ An end point method for this purpose^48^ determines the value of *ε* by comparing the amount of betalamic acid produced by alkaline hydrolysis of betanin [NH_4_OH_(aq)_, 1.2 mol L^–1^, *ε*(424 nm) = 65,000 L mol^–1^ cm^–1^][Bibr ref78] with the amount produced by an unknown betalain under identical experimental conditions. The absorption spectrum must be recorded immediately after preparing the working solution to avoid artifacts derived from the fast decomposition of betalains under acidic or alkaline conditions. This method assumes that betalamic acid is stable for the duration of the reaction,^48^ which was confirmed by our experiments (Figure 5 and Figures S1 – S3).

The main limitation of the hydrolysis approach (in addition to the uncertainties in the value of *ε* for betanin) is that not only structural modifications but changes in measurement conditions (e.g., pH, ionic strength, solvent, temperature) significantly alter the absorption properties of betalains.^89,90^ In fact, a noticeable change in the absorption maxima was observed for almost all betaxanthins studied when the pH was increased from 7.0 to 9.5 to accelerate hydrolysis (Figures S1 – S3). Consequently, the value of *ε* determined under alkaline conditions is not necessarily the same as that at pH 7. To determine the magnitude of this effect, the absorption and fluorescence spectra of Pro-Bx were obtained in BRB pH 7 to 11 (Figure 4b). There was a dramatic dependence of the spectral profile of Pro-Bx, especially the maximum absorption wavelength, on the pH. The same effect is not observed for betalamic acid, which maintains a nearly constant *ε* value over the 6 to 10 pH range (Figure 4a).

To address these limitations, we devised eq 1 in which the molar absorption coefficient for each betaxanthin was determined by following the kinetics of betalamic acid formation and betaxanthin hydrolysis (Figures S1 – S3). The kinetics was monitored at the wavelength of maximum absorption and pH 7 (*ε*’,Bx­(λ)). This approach provides insight into the reaction mechanism. Betaxanthin depletion and betalamic acid formation have matching observed rate constants (Figures S1 – S3, Table 1), despite some degree of spectral overlap, suggesting that hydrolysis is the main decomposition pathway under this experimental condition. Also, the absorption of betalamic acid reaches a plateau, confirming that it is not significantly decomposed during the experiment and enabling the estimation of the absorption at infinite time (A^HBt^_∞_) by nonlinear curve fitting, which is required for proper estimation of *ε*’,Bx­(λ). Use of *ε*(HBt, 424 nm) = 26,500 L mol^–1^ cm^–1^ ^58^ gave a mean ε of 53,000 L mol^–1^ cm^–1^ for amino acid betaxanthins, which is slightly higher than the value of 48,000 L mol^–1^ cm^–1^ previously reported.^32^

1 $$\varepsilon^{\prime ,\mathrm{Bx}}\left(\lambda \right)=26,500\frac{A^{\prime ,\mathrm{Bx}}\left(\lambda \right)}{A_{\infty }^{\mathrm{HBt}}\left(424\mathrm{nm}\right)}$$

### Fluorescence Spectra and Quantum Yields in Water

Fluorescence characterization can be complicated by the presence of impurities, especially in cases where the fluorescence quantum yield of the desired fluorophore is low.^91^ This highlights the necessity of considering synthetic and spectroscopic purity during fluorescence measurements. Using chromatographically pure betaxanthins,^55^ we have developed a comprehensive spectral database for this class of natural pigments, complementing existing databases for other important pigments classes, such as chlorophylls,^92^ tolyporphins,^93^ phyllobilins,^94^ and flavonoids.^95^ Making these data more accessible should not only enhance the understanding of biofluorescent organisms but also facilitate broader studies on fluorescent natural products.

The fluorescence of betaxanthins at around 508 nm (Figure 3) is distinctively different from that of betalamic acid (λ_Em_ = 483 nm, Figure 4a). The emission of Pro-Bx is slightly shifted to longer wavelengths compared to the other betaxanthins possibly due to the secondary nature of the precursor amino acid, which is expected to increase its charge-transfer character (Pro-Bx also has the longest absorption wavelength, at 486 nm). Trp-Bx is an exception with a ϕ_Fl_ of (3.3 ± 0.2) × 10^–4^; the low value may stem from the relatively low redox potential (E°(Trp­(−H^+^)^•^, H^+^/Trp) = 1.03 V and E°(Trp^•+^/Trp) = 1.24 V),^96^ which in turn promotes the deactivation of the excited state of Trp-Bx via photoinduced electron transfer.^97^ DA-Bx and Pyr-Bx, the decarboxylated pairs of DOPA-Bx and Pro-Bx, respectively, have blue-shifted absorption maxima of around 5 nm.

The effect of the carboxylic acid group on the absorption was also observed for the pair Lys-Bx and ε-Lys-Bx, since the maximum absorption of ε-Lys-Bx is slightly blue-shifted in relation to Lys-Bx. Pro-Bx and Pyr-Bx, which are the only secondary betaxanthins among the ones studied, showed emission maxima at slightly longer wavelengths than most betaxanthins, at 513 and 512 nm, respectively. These results are of particular interest for bioimaging applications given that betalamic acid can be used to functionalize proteins and thereby generate betaxanthin fluorescent labels.^53^ Betalamic acid reacts with nucleophiles to produce adducts; indeed, only betaxanthins derived from amines and amino acids have been found *in vivo*. Although Lys, Arg and His contain nitrogenous side chains, only the ε-amino group of Lys has been found to produce betaxanthins.^53,55^

### Loose Bolts and Fleeting Lives

The photochemistry of betaxanthins is limited by the short lifetime of the singlet excited state and low intersystem crossing efficiency because of ultrafast internal conversion via conical intersection.^23,26^ The possible occurrence of restricted conformational motion in certain environments can increase the value of ϕ_Fl_ and hence increase the practical brightness by suppressing internal conversion. Such relation was treated in 1939 by Lewis and Calvin,^98^ who wrote: *“loose bolts in some moving part of a machine ... provide a process by which the energy of the system is lost or degraded”*. The practical brightness is given by the product *ε* x ϕ_Fl_ for a given wavelength of absorption and detector window.^99^

Although the ϕ_Fl_ value of betaxanthins is rather low, the key determinant of photochemical processes is the lifetime of the excited state.^100^ Miraxanthin V and vulgaxanthin I undergo rapid excited-state deactivation in solution primarily through internal conversion rather than fluorescence, with lifetimes ranging from 2.9 to 37 ps depending on solvent conditions.^25,26^ Theoretical calculations suggest that betaxanthins exhibit weak fluorescence primarily due to a conical intersection between the S_1_ and S_0_ potential surfaces. Molecular torsion leads to ultrafast deactivation of the excited state, precluding the intersystem crossing to the triplet manifold.^23,26^ However, the size of these polymethine cyanines and the presence of many protomers and stereoisomers make the use of costly multireference and multiconfigurational methods challenging, as noted by Jacquemin and collaborators during their study of conventional cyanine dyes.^101^

The viscosity of the surrounding medium affects the S_1_ state lifetimes and fluorescence quantum yields. In ethylene glycol [viscosity at 25 °C (η) = 16.1 cP] internal rotations are hindered, leading to longer lifetimes and a 7-fold increase in ϕ_Fl_ (4.6 × 10^–3^ in water^24^ vs 3.3 × 10^–2^ in ethylene glycol^25–27^ ). To determine if a higher increase in viscosity had a more pronounced effect on the ϕ_Fl_ of Pro-Bx, a model betaxanthin (Figure 4c), we used a solution with 90% m/m of glycerol (Gl) in water [χ_Gl_ = 0.63; η­(25 °C) = 189 cP], which is >10-times more viscous than pure ethylene glycol. Interestingly, the ϕ_Fl_ of Pro-Bx in 90% Gl/W (4.6 × 10^–3^) was found to be only 1.4- or 10-times higher than that observed in ethylene glycol or water, respectively. The ϕ_Fl_ value was twice as high in 2,2,2-trifluoroethanol (TFE) compared to water (8.8 × 10^–3^ in TFE).^24^ Hence, viscosity effects seem to be more important for the fluorescence of betaxanthins than the change in medium polarity.

Structural modifications that restrict torsional movements can enhance fluorescence yields, suggesting that betaxanthins may be brighter in cellular environments because of environmental rigidity and specific interactions within the biological matrix.^21^ This behavior is similar to what has been observed with other polymethine dyes such as Cy3, where steric hindrance in rigid environments enhances the ϕ_Fl_ value by reducing nonradiative decay.^102^ The fact that betaxanthins are not photosensitizers whereas many polymethine cyanine dyes are^103^ highlights the potential application of betaxanthins as natural stains for cell biology studies.

The fluorescence quantum yield of betaxanthins in water can be close to 1%. However, this high fluorescence is often fleeting, as demonstrated by the rapid hydrolytic degradation of amino acid betaxanthins (Figure 5 and Figures S7–S10). This phenomenon underscores the challenge of balancing brightness with stability in fluorescent dyes. The hydrolytic stability of betaxanthins is a key consideration for their application in biological systems, particularly for imaging over extended periods. Under the experimental conditions employed in this study (phosphate buffer at pH 7, 50 °C), betaxanthins exhibit varying degrees of stability. His-Bx and Gly-Bx were found to be the least stable against hydrolysis, while Cys-Bx, Pyr-Bx, and Pro-Bx demonstrated greater resistance to degradation. For instance, after 6 h of testing, Cys-Bx showed only a 6% decrease in initial absorption, whereas most other derivatives decayed between 65% and 92%. This suggests that certain structural features may confer increased stability to these pigments.

Betaxanthins derived from amino acids can undergo decarboxylation. A mechanism for decarboxylation was first proposed by Dreiding.^104^ Decarboxylation appears to play a critical role in reducing fluorescence, as discussed by Gandia-Herrero and coauthors.^48^ Fluorescence intensity was enhanced by the presence of carboxylic groups and reduced by electron-donating groups; for example, DOPA-Bx and Pro-Bx display higher fluorescence than their decarboxylated counterparts DA-Bx and Pyr-Bx. Likewise, increasing the number of hydroxyl groups on the aromatic ring of Phe-Bx, Tyr-Bx, and DOPA-Bx resulted in a noticeable decrease in ϕ_Fl_, likely due to enhanced nonradiative decay pathways. This is relevant since betalain biosynthesis incorporates amino acids directly without decarboxylation, giving an acidic nature distinct from typical alkaline alkaloids.^105^ Although the term ″chromoalkaloids″ has been used to classify betalains, they have recently been categorized as a distinct class from alkaloids.^105^

DOPA-Bx and Pro-Bx have ϕ_Fl_ values higher than those determined for their decarboxylated pairs, DA-Bx and Pyr-Bx, respectively. Lys-Bx also has a higher ϕ_Fl_ value than its isomer, ε-Lys-Bx. No effect on ϕ_Fl_ was observed in the substitution of the carboxylic acid group for an amide in the amino acid side chain of Glu-Bx and Gln-Bx, for which similar ϕ_Fl_ values were determined. However, for the Asp-Bx and Asn-Bx pair, the substitution of the carboxylic acid by the amide group in Asn-Bx slightly increased the value of ϕ_Fl_ versus that determined for Asp-Bx. In addition, a decrease in the ϕ_Fl_ values was observed for Phe-Bx, Tyr-Bx and DOPA-Bx with an increase in the number of hydroxyl groups attached to the aromatic ring of these molecules.

The spectra for the betaxanthins described herein are available in PhotochemCAD, a software program with companion spectral databases for use in the photosciences.^56,57^ A particular focus has concerned databases of spectra of natural pigments; the databases are curated and contain references to the originating scientific literature. Databases assembled to date include spectra of chlorophylls and analogues,^92^ tolyporphins,^93^ phyllobilins,^94^ flavonoids,^95^ and bilins.^106^ The availability of the absorption and fluorescence spectra for betaxanthins should facilitate study of a class of colored natural products that heretofore has been under-appreciated.

## Outlook

Understanding the composition and function of natural pigments has been a central scientific concern for essentially two centuries. Betaxanthins are relatively recent newcomers to the well-studied plant pigments family. In this regard, the view of betalains has changed fundamentally over the years. Indeed, Sir Robert Robinson’s 1932 and 1933 papers^107,108^ were titled “Synthetical Experiments on the Nature of Betanin and Related Nitrogenous Anthocyanins,” yet by the 1960s betalains had come to be regarded as a family of pigments in their own right.^109^ Betaxanthins exhibit weak fluorescence in aqueous solution, are readily synthesized from diverse amines and betalamic acid, and are genetically encodable. Additionally, the nonradiative decay pathways of these compounds, particularly their internal conversion mechanisms, offer unique insights into how light energy is dissipated in plants, suggesting a photoprotective role for these pigments.

## Experimental Section

### General Experimental Procedures

Absorption spectra were acquired using a 10 mm path length quartz cuvette on a Cary 50 Bio spectrophotometer (Varian Inc., Mulgrave, VIC, Australia) equipped with a thermostated cell holder set at 25 ± 1 °C; 200–800 nm, a signal averaging time of 0.0125 s, a data interval of 1 nm, and a scan rate of 4,800 nm min^–1^. Fluorescence spectra were recorded using a 10 mm path length quartz cuvette on an FL980 spectrometer (Edinburgh Instruments, Livingston, UK) equipped with a cell holder thermostated at 25 ± 1 °C; emission (EM) wavelength interval: 450–700 nm, excitation (EX) wavelength (λ_EX_): 430 nm, EX and EM slits: 10 nm bandwidth, signal averaging time: 0.25 s, and data interval: 1 nm. All solvents and reagents were obtained from Sigma-Aldrich (St. Louis, MO, USA) and used without further purification. All solutions were prepared using deionized water (18.2 MΩ cm at 25 °C, TOC ≤ 4 ppb, Milli-Q, Millipore), Britton-Robinson buffer (BRB, pH 2–12, 40 mmol L^–1^)^110^ or water-glycerol mixtures. In all cases, no solvent absorbance or emission was detected under the experimental conditions used for spectral data acquisition. Since changes in the ionic strength of BRB with pH did not affect the spectroscopic properties or hydrolysis of betaxanthins, we did not adjust it with KCl.^111^ Results are expressed as mean ± standard deviation of triplicate experiments.

### Isolation of Betanin and Semisynthesis of Betaxanthins

Betanin was isolated from red beetroot juice by semipreparative reversed-phase HPLC, as described in detail elsewhere.^35^ Betaxanthins were prepared from betalamic acid.^55,59^ Structural characterization, including mass spectrometry and MS/MS fragmentation analysis, is provided in ref 55.

### Absorption Spectra and Estimation of Molar Absorption Coefficients

The molar absorption coefficient of each betaxanthin at the selected wavelength [*ε*′^,Bx^(λ), pH 7] was estimated from the absorption data [*A*′^,Bx^(λ), pH 7] using an end point method (eq 1), and is relative to that of betalamic acid (ε^424 nm^ = 26,500 L mol^–1^ cm^–1^).^58^ A stock solution of betaxanthin in water was used to prepare working solutions in Britton-Robinson buffer pH 7 and 9.5 using the same dilution factor. The alkaline hydrolysis of the betaxanthin at pH 9.5 (25 °C, BRB) was monitored spectrophotometrically until completion, i.e., the absorption of betalamic acid at 424 nm remains constant over time after 4 to 6 h. The absorbance of betalamic acid at infinite time (A^HBt^_∞_) was estimated by fitting an exponential function to kinetic traces obtained at 424 nm. The calculations assume stoichiometric conversion, and that betalamic acid is not decomposed during hydrolysis and its ε does not vary with the pH.

### Fluorescence Spectra and Determination of Fluorescence Quantum Yields

The fluorescence quantum yield (ϕ_Fl_) of each betaxanthin in air-equilibrated aqueous solutions (refractive index, *n*_D_ = 1.3329) was determined using eq 2; fluorescein in 0.1 mol L^–1^ aqueous NaOH (*n*_D_^r^ = 1.3325; ϕ_Fl_^r^ = 0.95) was used as the fluorescence standard.^112,113^ Stock solutions of each betaxanthin or fluorescein were used to prepare five solutions with absorption between 0.01 and 0.1. Fluorescence spectra of betaxanthins and fluorescein were acquired under identical experimental conditions, and the corresponding area under the curve (AUC) was calculated. The angular coefficients α and α^r^ were estimated by linear regression of AUC vs absorption at the excitation wavelength (430 nm) for betaxanthins and reference, respectively.

2 $$\phi_{\mathrm{Fl}}=\phi_{\mathrm{Fl}}^{r}\frac{\alpha }{\alpha^{r}}{\left(\frac{n_{D}}{n_{D}^{r}}\right)}^{2}$$

### Spectral Database

All spectra described herein can be accessed at no cost at www.photochemcad.com.

## Supplementary Material



## Notes

Raw data for the determination of molar absorption coefficients, fluorescence quantum yields, and hydrolytic stability (PDF)

## ∇

Cell Biology and Biophysics Unit, European Molecular Biology Laboratory, Heidelberg 69117 , Germany
