# Supplementary material for: Amino Acid Betaxanthins: Absorption, Fluorescence, And Stability
Source: J Nat Prod. 2025 Jun 20;88(10):2301–12. doi: 10.1021/acs.jnatprod.5c00419 (PMC12560082; doi:10.1021/acs.jnatprod.5c00419)
Supplement: Supplementary file 1 [file np5c00419_si_001.pdf]

SUPPORTING INFORMATION

Amino acid betaxanthins: absorption, fluorescence, and stability

Larissa C. Esteves,[a] Amanda C. Pinheiro,[a] Caroline O Machado,[a] Nathana B. L. Pettigiani,[a] Ana Clara B. Rodrigues,[a,‡] Masahiko Taniguchi,[b,*] Jonathan S. Lindsey,[b,*] and Erick L. Bastos[a,*]

[a] Department of Fundamental Chemistry, Institute of Chemistry, University of São Paulo, 05508-000 São Paulo, SP, Brazil.

[b] Department of Chemistry, North Carolina State University, Raleigh, NC 27607, USA.

‡ Current address: Cell Biology and Biophysics Unit, European Molecular Biology Laboratory, Heidelberg, Germany.

* Correspondence to: mtanigu@ncsu.edu (M. Taniguchi), jlindsey@ncsu.edu (J. S. Lindsey), and elbastos@usp.br (E. L. Bastos).

TABLE OF CONTENTS

1. Molar absorption coefficient S2

2. Fluorescence quantum yield S6

3. Hydrolytic stability S9

1. Molar absorption coefficient

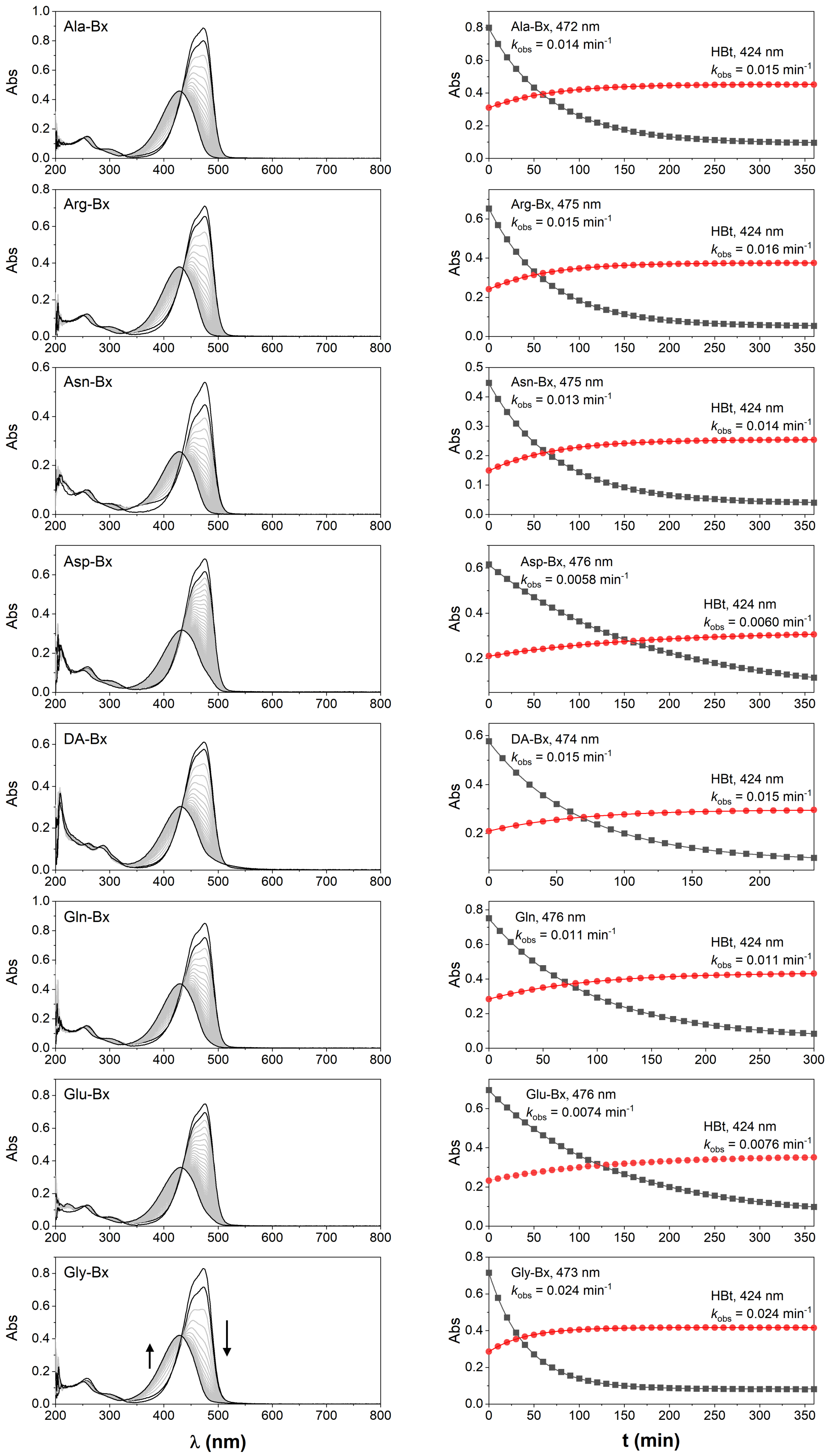

Figure S1. Hydrolysis of Ala-Bx, Arg-Bx, Asn-Bx, Asp-Bx, DA-Bx, Gln-Bx, Glu-Bx, and Gly-Bx in Britton-Robinson buffer (40 mmol L^{-1}, pH 9.5), and the correspondent kinetics of betalamic acid formation (HBt, λ = 424 nm) and betaxanthin degradation (λ ≈ 475 nm). The spectrum with the highest intensity corresponds to the one obtained at pH 7, representing the initial absorption of betaxanthins.

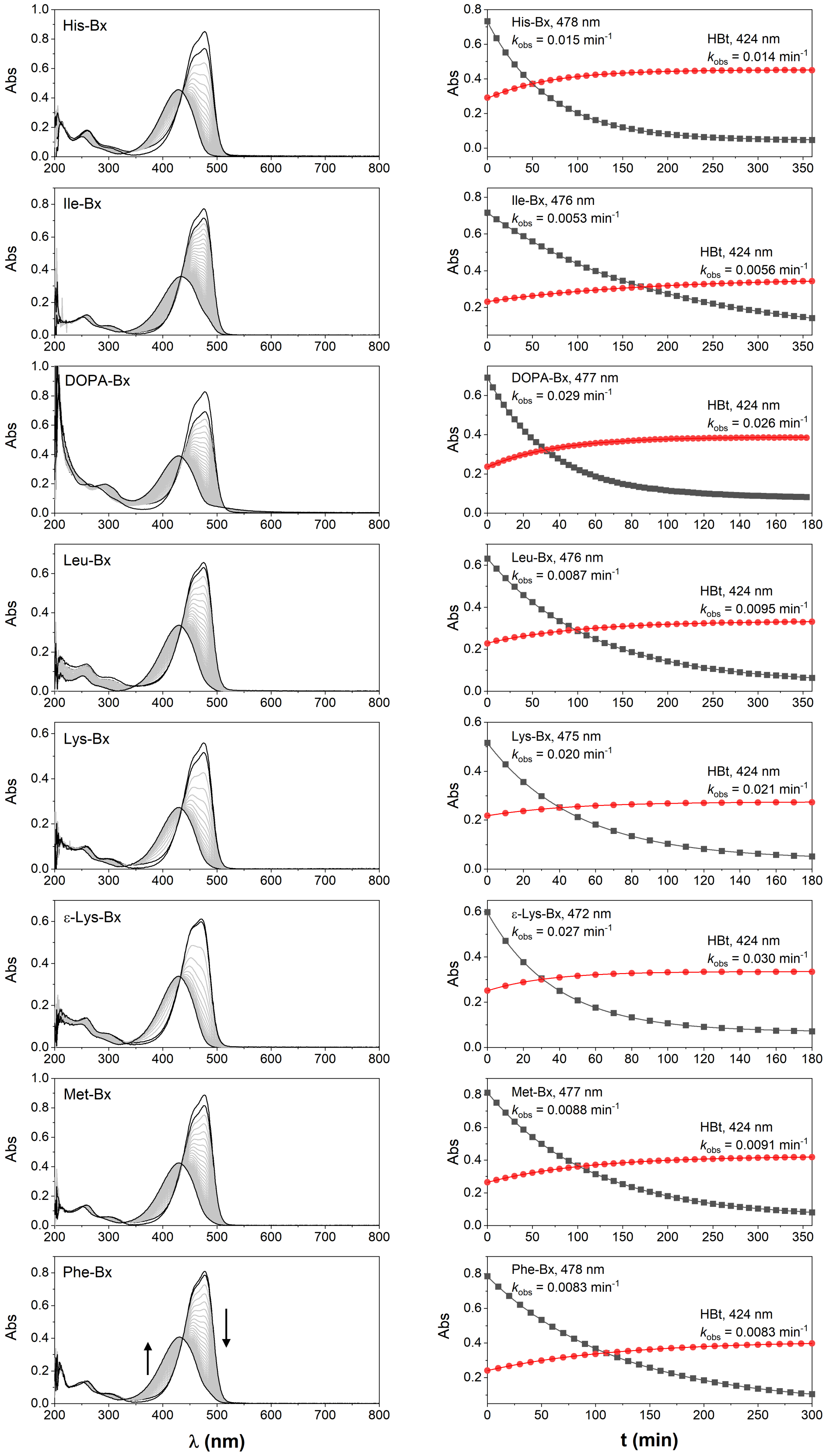

Figure S2. Hydrolysis of His-Bx, Ile-Bx, DOPA-Bx, Leu-Bx, Lys-Bx, ε-Lys-Bx, Met-Bx, and Phe-Bx in Britton-Robinson buffer (40 mmol L^{-1}, pH 9.5), and the correspondent kinetics of betalamic acid formation (HBt, λ = 424 nm) and betaxanthin degradation (λ ≈ 475 nm). The spectrum with the highest intensity corresponds to the one obtained at pH 7, representing the initial absorption of betaxanthins.

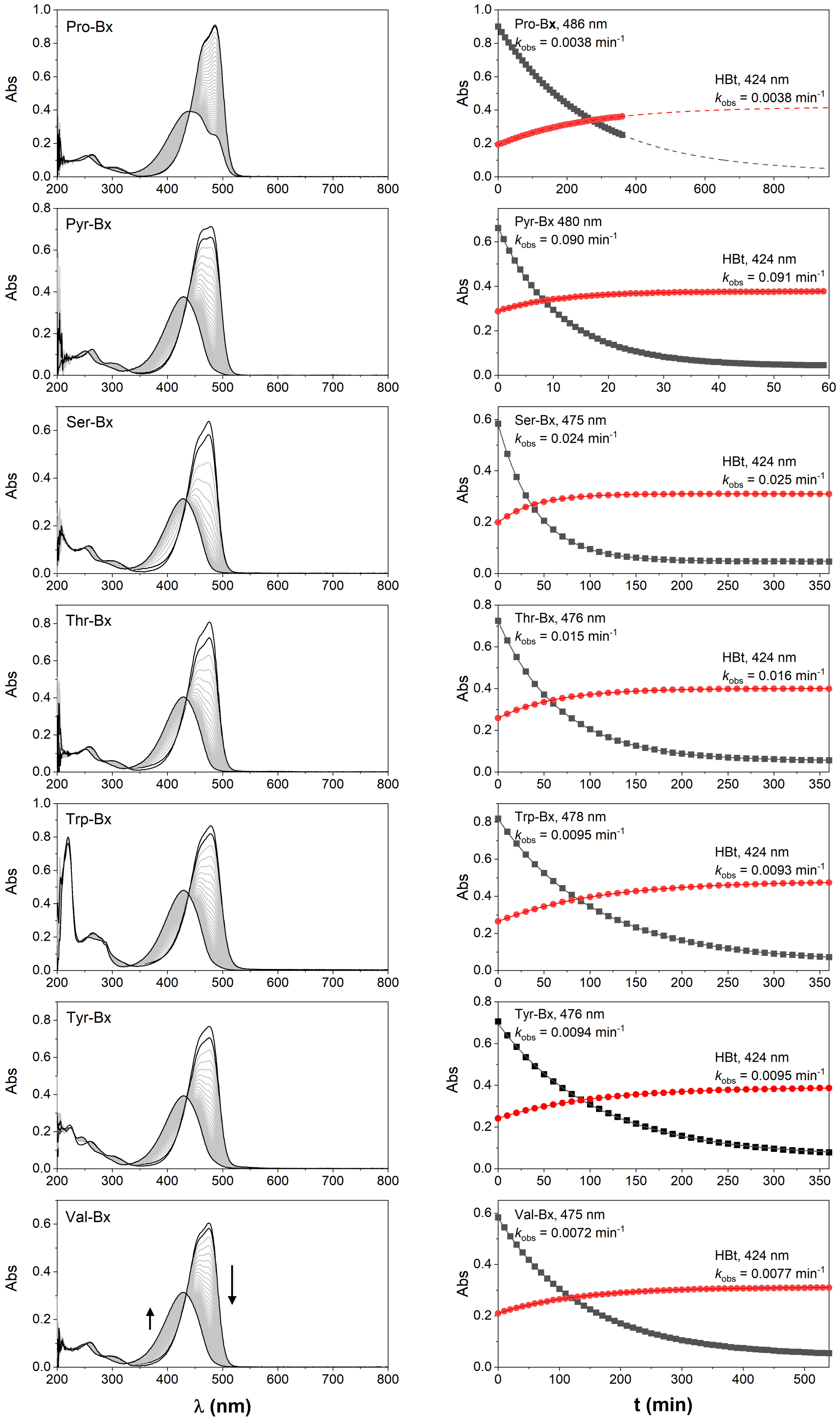

Figure S3. Hydrolysis of Pro-Bx, Ser-Bx, Thr-Bx, Trp-Bx, Tyr-Bx, and Val-Bx in Britton-Robinson buffer (40 mmol L^{-1}, pH 9.5), and of Pyr-Bx in Britton-Robinson buffer (40 mmol L^{-1}, pH 10.5), along with the corresponding kinetics of betalamic acid (HBt) formation (λ = 424 nm) and betaxanthin degradation ($\lambda \approx$ 475 nm). The spectrum with the highest intensity corresponds to the one obtained at pH 7, representing the initial absorption of betaxanthins.

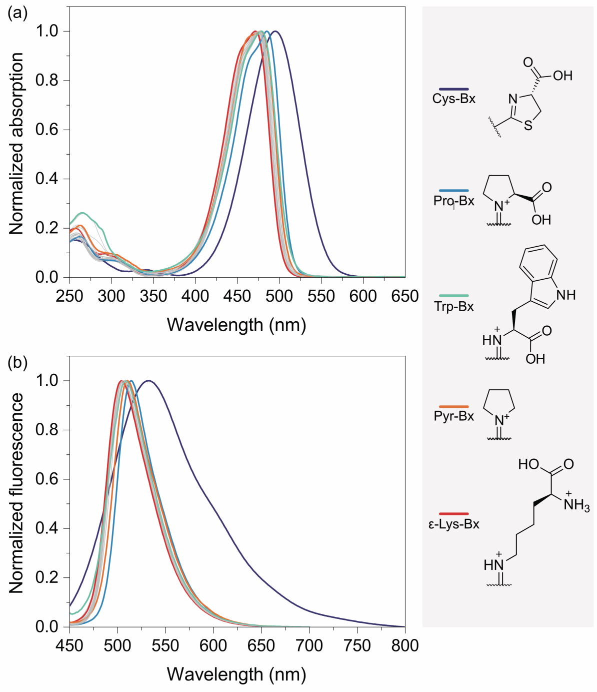

Figure S4. Comparison of normalized absorption (a) and fluorescence (b) spectra of betaxanthins synthesized from amino acids, pyrrolidine, or dopamine. Representative examples are highlighted in color.

2. Fluorescence quantum yield

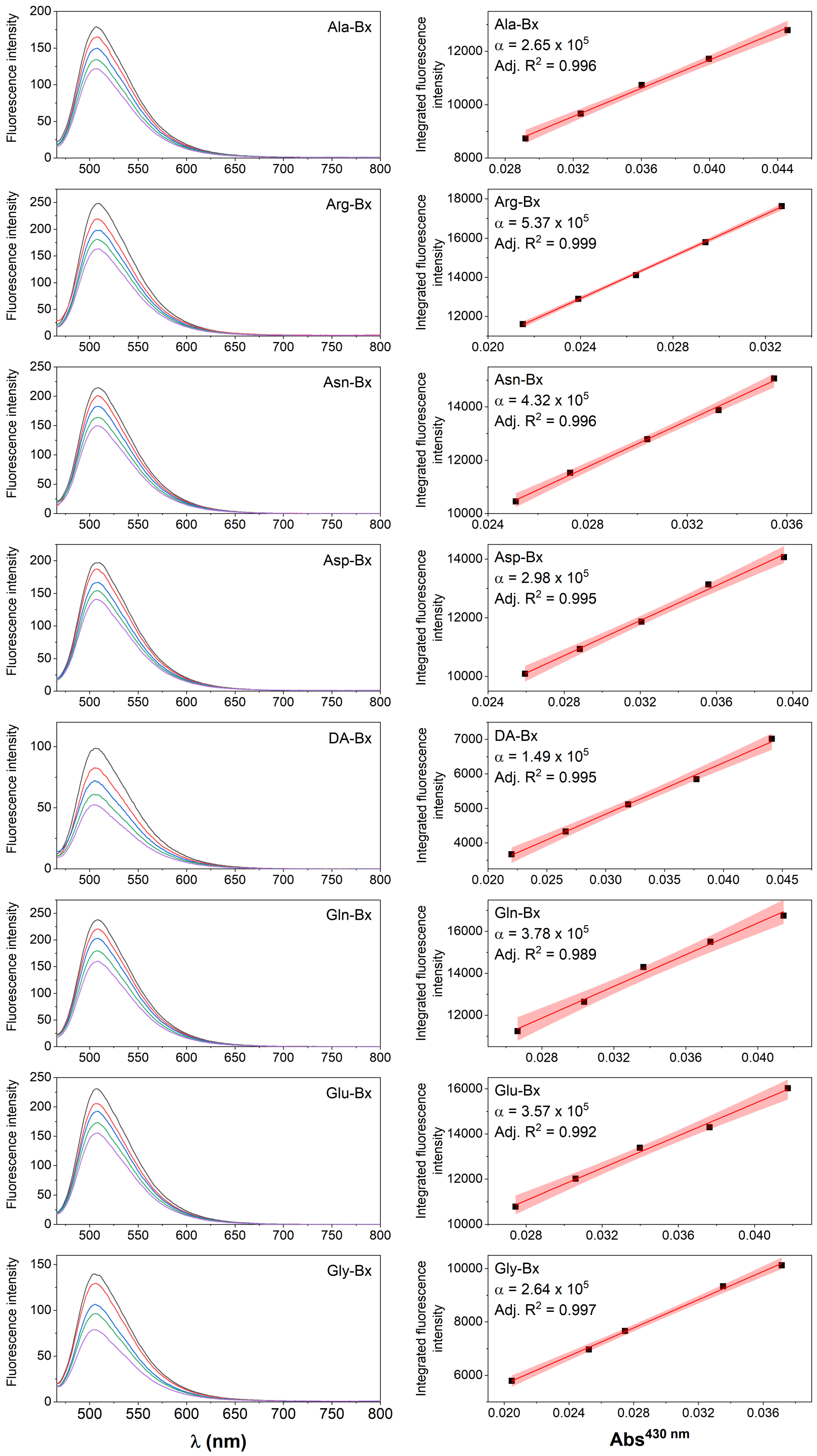

Figure S5. Fluorescence emission spectra of Ala-Bx, Arg-Bx, Asn-Bx, Asp-Bx, DA-Bx, Gln-Bx, Glu-Bx, and Gly-Bx (λ^{EX} = 430 nm, in water), and corresponding linear correlations between emission area and absorbance.

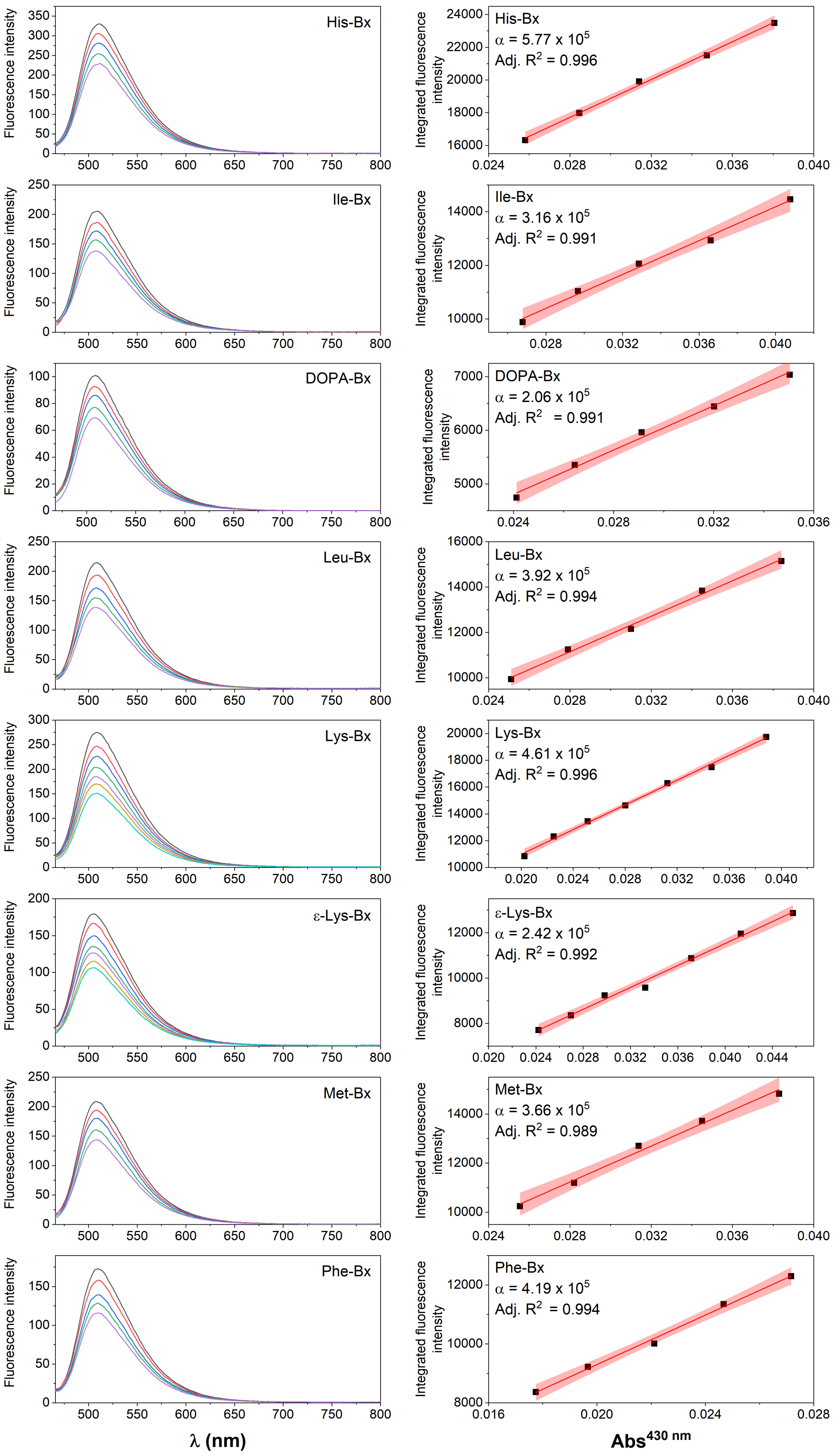

Figure S6. Fluorescence emission spectra of His-Bx, Ile-Bx, DOPA-Bx, Leu-Bx, Lys-Bx, ε-Lys-Bx, Met-Bx, and Phe-Bx (λ^{EX} = 430 nm, in water), and corresponding linear correlations between emission area and absorbance.

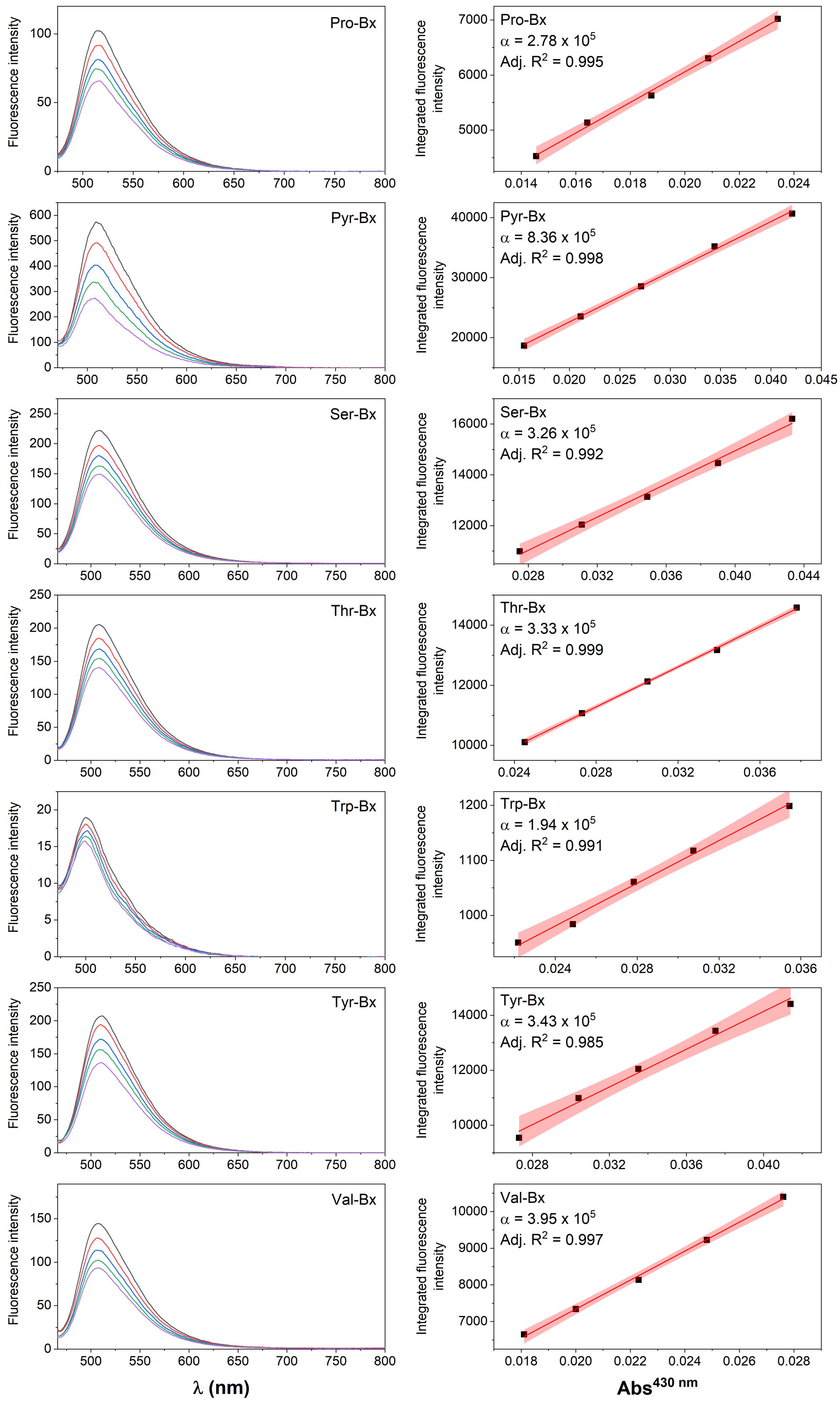

Figure S7. Fluorescence emission spectra of Pro-Bx, Pyr-Bx, Ser-Bx, Thr-Bx, Trp-Bx, Tyr-Bx, and Val-Bx (λ^{EX} = 430 nm, in water), and corresponding linear correlations between emission area and absorbance.

3. Hydrolytic stability

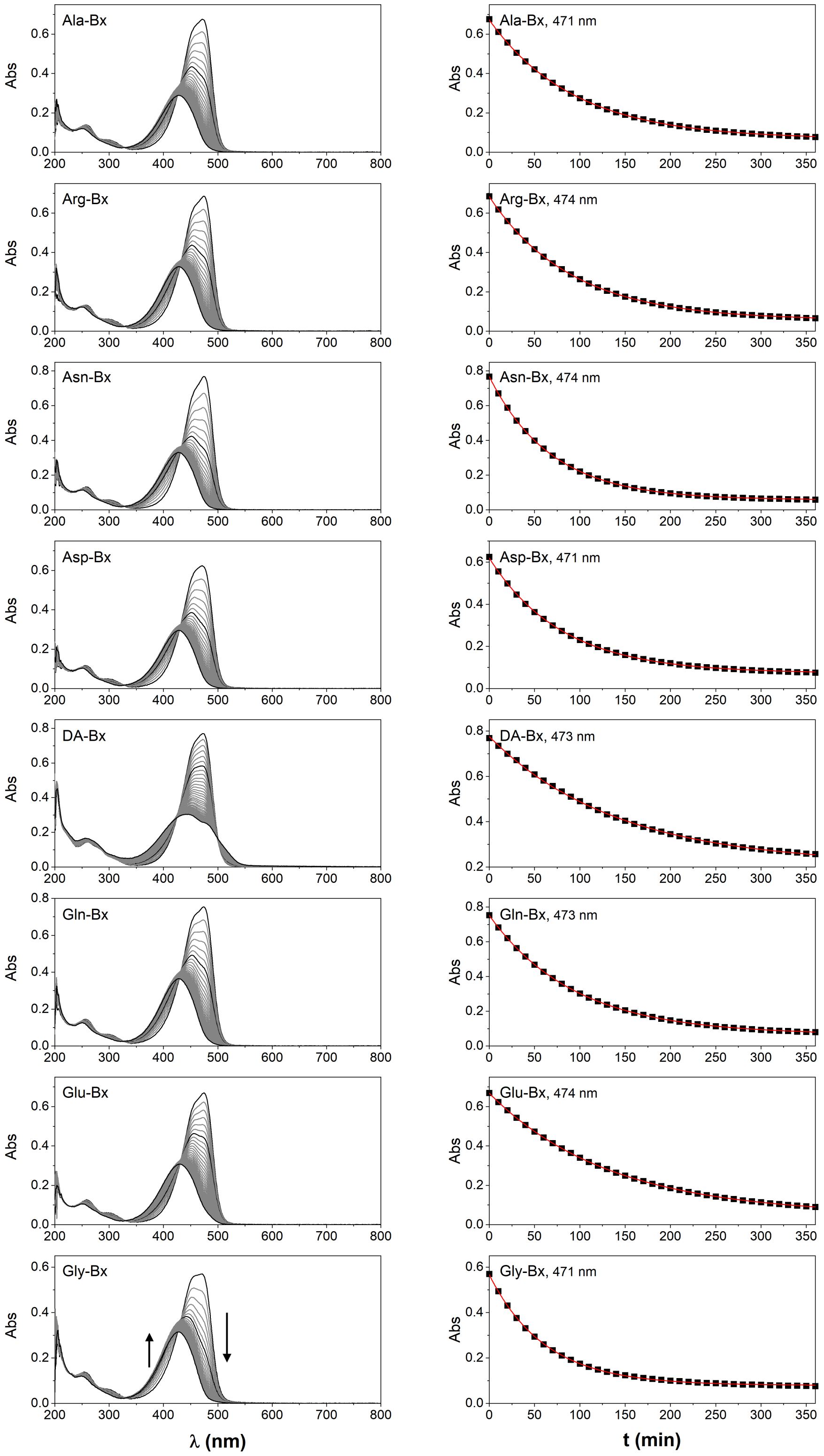

Figure S8. Hydrolysis of Ala-Bx, Arg-Bx, Asn-Bx, Asp-Bx, DA-Bx, Gln-Bx, Glu-Bx, and Gly-Bx in phosphate buffer (100 mmol L^{-1}, pH 7) at 50 °C, with corresponding monoexponential fits of the absorption data at the betaxanthin absorption maximum as a function of time. Arrows indicate the decrease in betaxanthin absorption ($\lambda \approx 475$ nm) and the increase in betalamic acid (HBt) absorption ($\lambda \approx 428$ nm). Spectra highlighted in black correspond to time points t_0, $t = 1$ h, and $t = 6$ h.

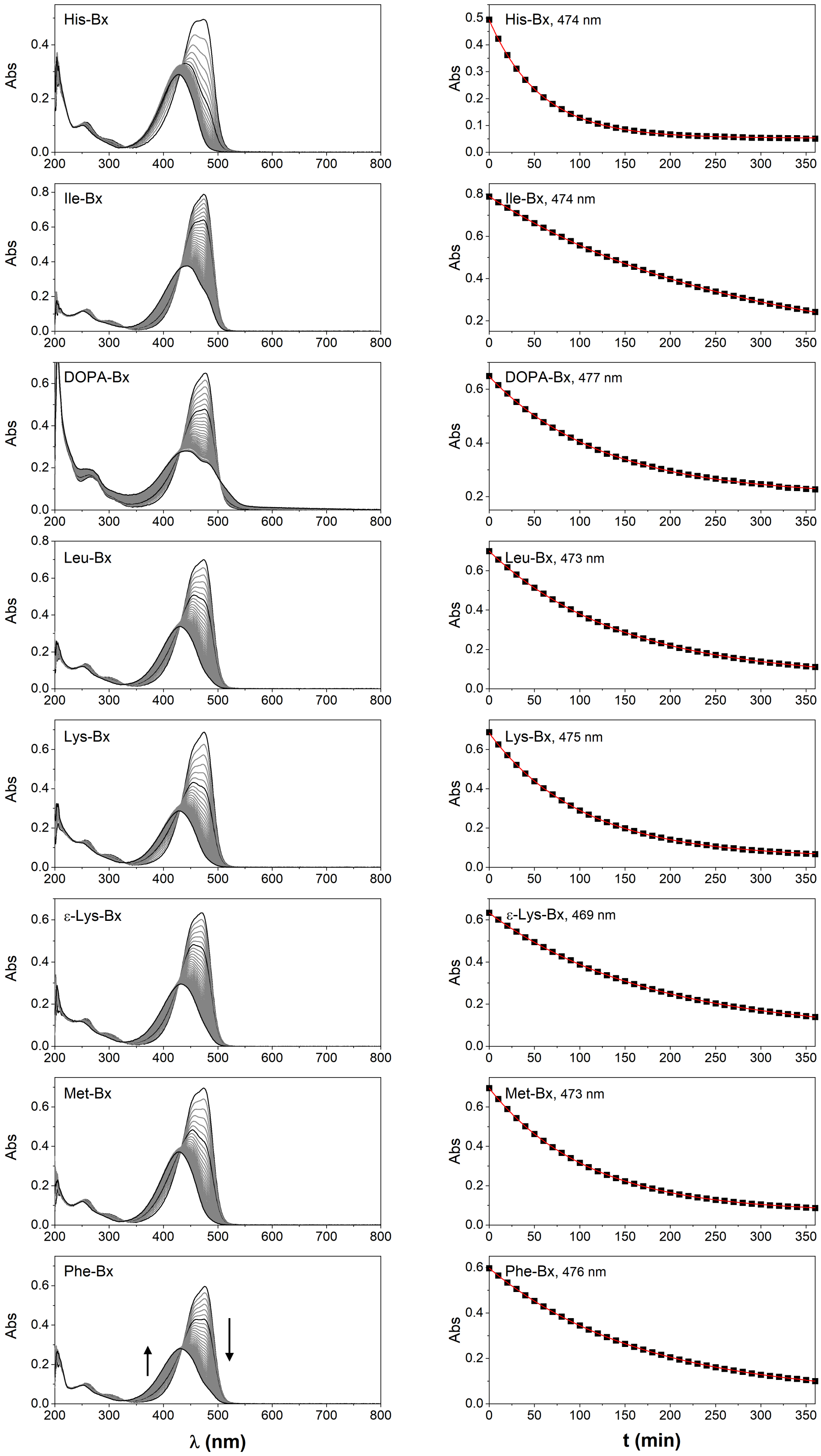

Figure S9. Hydrolysis of His-Bx, Ile-Bx, DOPA-Bx, Leu-Bx, Lys-Bx, ε-Lys-Bx, Met-Bx, and Phe-Bx in phosphate buffer (100 mmol L^{-1}, pH 7) at 50 °C, with corresponding monoexponential fits of the absorption data at the betaxanthin absorption maximum as a function of time. Arrows indicate the decrease in betaxanthin absorption ($\lambda \approx 475$ nm) and the increase in betalamic acid (HBt) absorption ($\lambda \approx 428$ nm). Spectra highlighted in black correspond to time points t_0, $t = 1$ h, and $t = 6$ h.

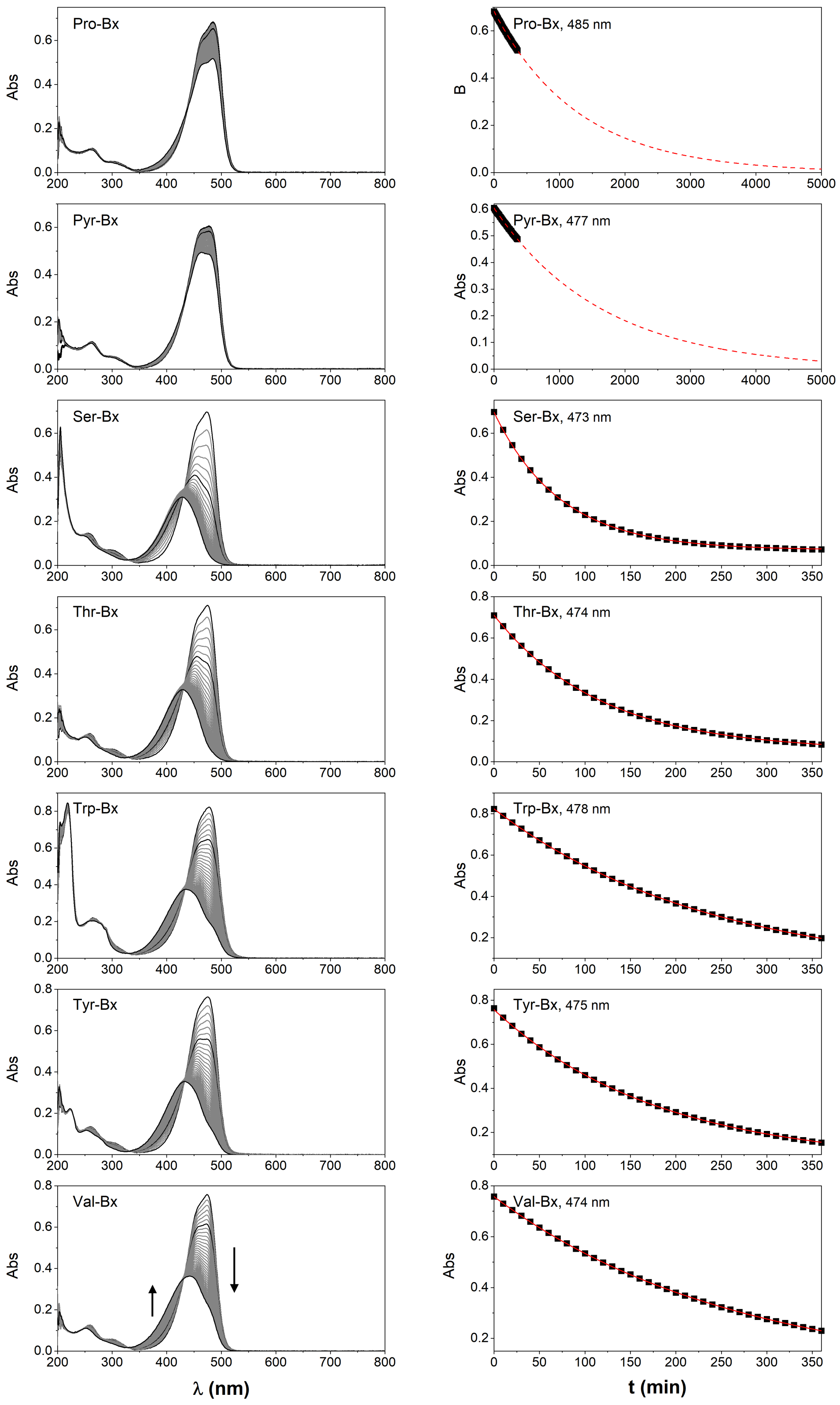

Figure S10. Hydrolysis of Pro-Bx, Pyr-Bx, Ser-Bx, Thr-Bx, Trp-Bx, Tyr-Bx, and Val-Bx in phosphate buffer (100 mmol L^{-1}, pH 7) at 50 °C, with corresponding monoexponential fits of the absorption data at the betaxanthin absorption maximum as a function of time. Arrows indicate the decrease in betaxanthin absorption (λ ≈ 475 nm) and the increase in betalamic acid (HBt) absorption (λ ≈ 428 nm). Spectra highlighted in black correspond to time points t_0, t = 1 h, and t = 6 h.

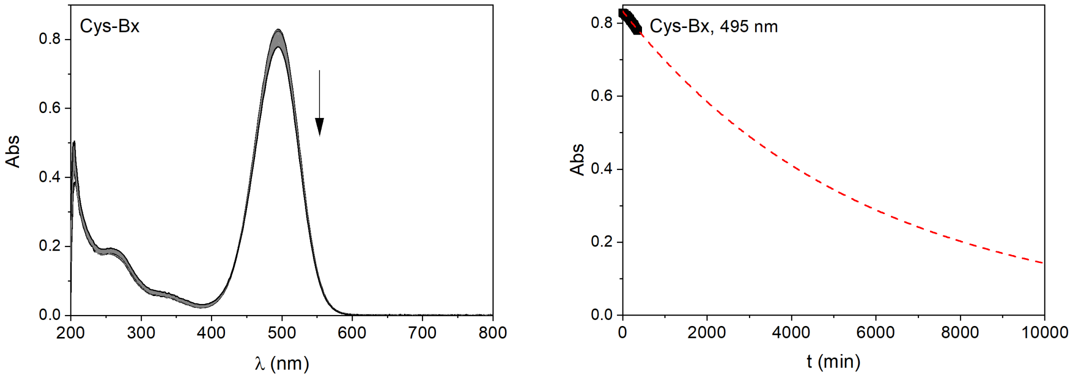

Figure S11. Hydrolysis of Cys-Bx in phosphate buffer (100 mmol L^{-1}, pH 7) at 50 °C, with corresponding monoexponential fits of the absorption data at the betaxanthin absorption maximum as a function of time. Arrows indicate the decrease in betaxanthin absorption (λ = 495 nm). Spectra highlighted in black correspond to time points t_0, t = 1 h, and t = 6 h.
